# The Evolution, Current Indications and Outcomes of Cementless Total Knee Arthroplasty

**DOI:** 10.3390/jcm11226608

**Published:** 2022-11-08

**Authors:** Maria T. Schwabe, Charles P. Hannon

**Affiliations:** Department of Orthopaedics, Washington University in St. Louis, St. Louis, MO 63110, USA

**Keywords:** cementless, total knee arthroplasty, knee replacement, knee osteoarthritis

## Abstract

Total knee arthroplasty (TKA) has been performed by orthopedic surgeons for decades, but the cementless TKA has only recently gained much interest in the world of arthroplasty. Initially, early designs had multiple complications, particularly with aseptic loosening due to osteolysis and micromotion. However, modifications have shown good outcomes and excellent survivorship. Over the last several decades, changes in implant designs as well as implant materials/coatings have helped with bone in growth and stability. Furthermore, surgeons have been performing TKA in younger and more obese patients as these populations have been increasing. Good results from the cementless TKA compared to cemented TKA may be a better option in these more challenging populations, as several studies have shown greater survivorship in patients that are younger and have a greater BMI. Additionally, a cementless TKA may be more cost effective, which remains a concern in today’s healthcare environment. Overall, cemented and cementless TKA have great results in modern times and there is still a debate as to which implant is superior.

## 1. Introduction

Total knee arthroplasty (TKA) has been performed by orthopedic surgeons for decades with the first foundational design developed in the 1970s. The goal was to create an anatomic and functional knee that would survive many years [1]. Fifty years later, about 1 million TKAs are now performed annually in the United States, with projections to be 3.48 million in 2030 [2]. This procedure has reliably provided pain relief and excellent functional outcomes. However, there are concerns regarding the long-term survivorship especially with the rise of TKA in younger, more active, and more obese patients [3,4].

Traditionally, TKA has been fixed with cement for the femoral, tibial and when indicated, patella components. A majority of studies looking at survivorship have evaluated cemented TKAs. The advantages of cemented TKA are the more reliable fixation particularly in patients with poor bone quality, the ability to help fill any defects or compensate for inaccurate bone cuts, or the opportunity to include antibiotics in cement to help mitigate the risk of infection [5,6,7,8]. While survivorship of cemented TKAs has been good at 20 years, aseptic loosening is still common. Several studies have shown moderate rates of aseptic loosening at the bone cement interface as well as implant debonding at the implant cement interface [9,10]. Other common modes of failure include infection, arthrofibrosis, polyethylene wear, malalignment, and extensor lag [11]. To help mitigate this risk of aseptic loosening there has been a longstanding interest in cementless fixation for TKA.

In cementless TKA, the goal is for biologic fixation and ultimately improved survivorship. With the rise of TKA in younger, more active and more obese patients, the number of cementless TKAs performed has grown as well [3,4]. For instance, use of cementless TKA in male patients younger than 65 has shown a significant amount of less revisions than cemented TKA, according to American Joint Replacement Registry Annual Report 2020 [12]. Additionally, studies showing shorter operative time, less blood loss, and possibly less overall cost have further peaked interest in cementless TKA [13,14]. With these promising results, could cementless TKA become equivalent to or even be superior to the gold standard cemented TKA? Are there certain instances where the cementless TKA should be used as opposed to the cemented TKA? This review discusses the evolution, current indications and modern day outcomes of cementless TKA.

## 2. Evolution of Cementless TKA

The first cementless condylar TKA design was in the 1970s by Kodama-Yamamoto, but did not gain much traction until the 1980s [15]. The goal was to create a design that preserved bone stock and allowed for permanent fixation through osseointegration. Multiple different implant manufacturers produced a cementless TKA, but these first-generation TKA designs ultimately high rates of early failure. The most common complications of the early cementless TKA included aseptic loosening due to micromotion and poor fixation [16,17]. This was commonly seen at the tibial baseplate. Additionally, failure of the metal backed patella component was also not uncommon and led to another common reason for revision surgery [18]. Over several decades, changes in implant material, design and geometry were made to improve the overall function and long-term durability of the cementless TKA.

### 2.1. Implant Material

In the initial designs, there was quite a variation in component material as well as coating to support fixation. The first designs of Kodama-Yamamoto used stainless steel for the femoral component and polyethylene for the tibial component. Later generations used cobalt chrome given its increased wear resistance and stiffness [15].

In early generations, coating was added to improve fixation. Porous coating was the standard, but patch porous coating was also used to improve survivability. Unfortunately, polyethylene debris, osteolysis, and loosening were not uncommon with these patch porous designs and led to poor outcomes. A comparative study of the Ortholoc II (Wright Medical Technology, Arlington, TN, USA) (fully porous) showed 0/675 with radiographic lucency around the stem or pegs, only 27 with partial tibial lucency and only 1 with complete tibial lucency [19]. Of the Ortholoc Modular long-stem (patch porous), 28/124 had long stem lucency, 15 had partial tibial lucency, and 4 had complete tibial lucency [19]. The Ortholoc Modular short-stem showed similar results, demonstrating inferior radiographic outcomes in the patch porous implants [19].

Hydroxyapatite (Ca_10_[PO_4_]_6_[OH]_2_) coating was another major evolution in overall composition of the implant. Introduced to arthroplasty in the 1980s, this bioceramic helps to accelerate bone ingrowth through its inherent osteoconductive properties [20,21]. With these properties, the goal is to diminish micromotion in cementless TKA and multiple studies have supported this [22,23]. Carlsson et al. (2005) utilized radiostereometry to compare cemented, cementless porous, and cementless porous with hydroxyapatite coating. At 5 years, one revision occurred in each cementless group but less motion was observed in the hydroxyapatite group [24].

More recently, porous metals have been utilized including, but not limited to trabecular metal, porous titanium, tritanium, BioFoam and Regenerex^®^ to enhance the press-fit. These porous metals have porosity 60–65% porosity and a rougher surface, which maximizes initial press-fit, minimizes micromotion, and allows for biologic fixation [25]. In addition the porous coating is more commonly under the baseplate only which prevents stress shielding.

Several studies have evaluated cementless fixation with these porous metals. Pulido et al. (2015) performed a randomized controlled trial comparing cemented traditional, cemented trabecular, and uncemented trabecular TKA. Survivorship at 5 years was 95.3%, 96.5% and 97.2%, respectively [26]. However, several other studies demonstrated early failure rates with trabecular metals but more studies are still needed [27,28]. BIOFOAM, a cancellous and porous titanium coating seen in Wright Medical Technology’s ADVANCE knee system, was created to reduce stress shielding by having more resemblance to native bone properties. At 24 month follow-up, of 104 TKAs, only 2 participants required a revision and only one participant had radiographic evidence of tibial component lucency [29].

Today, a majority of cementless TKA implants are made with porous plasma sprayed (PPS) tibia or 3D printing. The Anatomic Graduated Component (AGC, Biomet, Warsaw, IN, USA) utilized plasma spray in the tibial component and studies showed a 97% survival rate of this component at 20 years [30,31]. Winther et al. (2016) investigated plasma spray further by comparing Vanguard PPS versus Regenerex^®^, a titanium based tibial component, both Biomet designs (Warsaw, IN, USA). They used radiostereometric analysis and found the PPS tibia had significantly less subsidence at 3 months but no significant difference was seen between the two groups at 24 months, indicating that the well-established PPS coated tibia remains a reliable implant [32]. Recently, a similar randomized controlled study was performed comparing these two groups and again, found no significant difference in micromotion of these groups at 60 months but longer follow-up results are still pending [33].

3D printing has further enhanced cementless total knee replacement with highly porous surfaces and optimized component designs that minimize micromotion. The few studies performed have shown increased osteointegration, less stress shield and delayed aseptic loosening [13,34,35,36]. Early follow-up at 6 and 12 weeks have shown more micromotion in the cementless TKA compared to the cemented TKA. However, tibial migration does appear to stabilize beyond this short-term follow-up [37,38,39]. Several studies have evaluated mid to long term follow-up of implementation 3D printed cementless TKA implants. One study has evaluated mid-term outcomes. Restrepo et al. (2021) reviewed patient reported outcomes, aseptic loosening and revision rates in the cementless Triathlon Tritanium 3D printed implant in 374 TKAs (Stryker Orthopaedics, Mahwah, NJ, USA). At a mean follow-up of 5.5 years, there were 11 (2.94%) revisions with 6 (1.6%) attributed to aseptic loosening and clinical scores significantly improved post-operatively [40].

### 2.2. Implant Design

Cementless TKA designs have also changed. In the first generation cementless TKA, the tibial component was the leading cause of failure. This was often due to poor initial fixation with many of the earlier designs lacking a keel or stem and instead using pegs. This led to catastrophic failure [41,42]. Similar issues were found with femoral components with fractures, lucency at the anterior flange and lucency at the posterior condyles of the femoral component, as well as abnormal patellofemoral tracking [43,44,45].

The initial modification to the tibial components included the addition of screws in the baseplate and stems to increase initial fixation and limit micromotion. The Miller-Galante I TKA (Zimmer, Warsaw, IN, USA) was developed in the 1980s and had 4 screws in the tibial baseplate. A metal-backed patella component was also initially used, but this was eventually converted to all polyethylene. The Miller Galante I and II prosthesis showed problematic lucency around the screw bone interface as well as cystic changes in the proximal tibia at short term follow-up [46]. In addition, at an average of 11 year follow-up, 48% of metal-backed patella components were revised and 9% tibial components had aseptic loosening, primarily due to lucency around the screws [18]. Similarly, the Arizona (Depuy, Warsaw, IN, USA) and Synatomic prosthesis (Depuy), which involved 4 screws and initially a polyethylene tibial component had a poor revision rate of 56% at an average of 45 months, primarily due to loosening around the medial tibial component as well as extension of osteolysis distally along the bone screw interface [17]. In 1985, the Natural Knee System was designed with 4 pegs and an optional cancellous screw for the central keel. Instead of a metal-backed patella, a stepped anterior chamber cut allowed for a more anatomic patellofemoral joint and motion [47]. The 1985 Press-Fit Condylar Knee (PFC; Johnson and Johnson, Raynham, MA, USA) utilized a finned tibial component and the cementless group had excellent results at an average of 2.8 years, with clinically similar Knee Society Scoring System results and no difference in radiolucent lines comparted to the cemented TKA [48]. However, at long term follow-up, aseptic loosening was found in 72% in the cementless group compared to 94% in the cemented group. As in the Miller-Galante total knee system, the metal backed patellar component also caused much failure [49].

With high rates of early failure, cementless TKA did not gain much traction until more modern designs were introduced in the beginning of the 21st century such as the Attune (Depuy, Warsaw, IN, USA), Triathlon (Stryker), or NexGen (Zimmer Biomet, Warsaw, IN, USA) TKA systems. Modern designs evolved to include keels and pegs that provide better initial rotational and axial stability, and ultimately, better survivorship. A systematic review of Triathlon cementless TKA had 98.7% survivorship and a mean KSS 90.2 at a range of 1.8–8 year follow-up time frame. Most notable, only 0.8% had aseptic loosening [50]. Additionally, rotating platform TKA appeared to enhance overall component relationship and kinematics. Ali and Mangaleshkar (2006) showed 99% survivorship of the rotating platform at 10 years [51]. Lastly, although severely lacking in studies, robotic assisted TKA may be another option to improve the results of cementless implants with theoretical more precision in the bone preparation that will allow for improved fixation [52].

A continued debate revolves around the patella component. With such early complications and difficulties with patella resurfacing, especially cementless, there is still a question of whether or not to even resurface the patella. Initially, in the 1980s and 1990s, the metal-backed patella was a common source of complication and revisions, due to fractures, dislocations, aseptic loosening, osteolysis and extensor mechanism disruption [53,54]. Up to 50% of revisions were secondary to the patellofemoral joint [55]. The Food and Drug Administration noted it to be the leading cause of failure in early TKA [56]. When it failed, removal and revision of the patella was challenging, as many of these components had ingrown pegs and surrounding bone loss [57]. Therefore, design modifications were made due these high failure rates. The Miller-Galante I design, with a dome-shaped metal-backed patella, was compared to the Miller-Galante II design, with a modified dome-shaped metal-backed patella, and found an increased in cumulative survival rates at 9 years, from 73% to 93%, respectively [58]. Additionally, the mobile bearing arthroplasty was created to allow for improved motion, which was first developed as early as the late 1970s and seen in the New Jersey Low Contact Stress system (LCS; Depuy, Inc, Warsaw, IN, USA). The focus was to create better congruity and thus, distribution of contact stresses and less wear debris. Specifically, at long-term follow-up, the rotating-bearing TKA only led to one failure of the patella (0.6%) requiring revision, which was due to metallosis and osteolysis [59]. More modern implants have the advantage of improved polyethylene and metal backing interfaces, better biologic coatings, and thicker pegs to help with cementless patella resurfacing. Grau et al. (2021) showed 100% aseptic survivorship at 5 years of the patella component in the 3D printed Stryker Triathlon Tritanium cementless patella. Additionally, only 6.2% of the patella components had radiolucent lines at 2 years [60]. Still, whether or not to resurface the patella in cementless TKA remains to be debated amongst surgeons today.

## 3. Indication Considerations

With newer and more modern designs, surgeons are more willing to utilize the cementless TKA. Moreover, cementless TKA has advantages in certain populations, such as in the younger and larger patient. These two populations are also becoming more common and more in need of a TKA. However, there are some concerns utilizing cementless TKA implants in patients with poor bone quality such as patients with osteoporosis or rheumatoid arthritis.

### 3.1. Young Age

Studies have demonstrated the growing number of young patients who will be in need of a TKA. Kurtz et al. (2006) projected that the demand for TKA would grow from 59,077 in 2006 to 994,104 in 2030 in those aged 45 to 54 years old [3]. However, young age has been associated with a higher revision rate, even after adjustment for sex, diagnosis, patellar resurfacing, TKA design, and fixation type [61].

One of the first studies focusing on the younger patient was performed in the 1990s with one of the first generation implants, the Profix (Smith and Nephew, Memphis, TN, USA). A comparative study of younger (<55 years old), larger patients and older, lighter patients was performed with a minimum of 5 year follow-up. No patient had a revision due to aseptic loosening and the only revision performed was due to polyethylene wear in the younger patient group. After 5 years, KSS was 88 and 92 in the younger and older population, respectively, and pain and function scores greatly improved in both groups. However, many patients in both groups had clinically asymptomatic radiolucent lines by one year, particularly around the anterior flange [62]. Similarly, 108 knees from 1977–1992 and aged 22–55 years old were analyzed and results showed only 2 infections, 1 tibial revision, 3 patella revisions and a spacer exchange. Non-progressive tibial lucency was seen in 9%, consistent with prior studies. Again, clinical results were excellent, even in these first generation implants [63]. Even with these promising results, these initial protheses had risk of aseptic loosening and one of the major concerns of early studies in the young was the need for polyethylene exchange due to wear and instability, likely due to the higher demand [64]. More recently, studies began to look at more modern implants and outcomes in young patients, with some reaching 100% survivorship at a mean follow-up of 4 years. [65]. A meta-analysis of randomized controlled studies from 2009 to 2016 compared cemented and cementless TKA in a younger population. No significant difference existed amongst Knee Society Score, aseptic loosening, motion or complications but cementless TKA had significantly better radiological outcomes and less pain [66]. With these results, the cementless TKA may be preferred in younger patients who have good bone quality and high activity.

### 3.2. Obesity

Another population of great interest and in need of a TKA is the obese population. Cemented fixation in TKA in patients with an elevated BMI can have at least 2–3× the revision rate than non-obese patients [67,68]. This is likely caused by micromotion at the bone cement interface as evidenced by increased aseptic loosening, focal osteolysis and radiographic lucency [69]. Additionally, obese patients have a significantly higher odds of infection and revision and a trend towards worse outcome scores [70]. This is particularly concerning as the obese patients are one of the largest growing populations in need of TKA [4]. Therefore, studies have investigated cementless TKA as a possibly better option. At an average follow-up of about 5 years, a direct comparison of normal weight, overweight, moderately obese and severely obese patients showed no significant difference in complications, revisions or 10-year survival of a posterior-stabilized, rotating platform cementless TKA between each group of patients. As expected, function and motion did decrease as obesity BMI increased [71]. Boyle et al. (2018) and Goh et al. (2022) found no significant difference in clinical outcomes, radiographs or survivorship in cemented versus cementless TKA in patients with an elevated BMI [72,73]. However, Bagsy et al. (2016) showed less revisions and less aseptic loosening in cementless TKA compared to cemented TKA in obese patients [74]. Additionally, Sinicrope et al. (2019) showed 99.1% survivorship of aseptic loosening in cementless TKA in patients with a BMI ≥ 40 at 8 year follow-up compared to 88.2% in the cemented group [75]. This may be due to the osseointegration of the implant and ability to tolerate stress shielding better than cemented TKA.

### 3.3. Rheumatoid Arthritis

Several studies have evaluated cementless TKA specifically in rheumatoid arthritis (RA) patients, who have poor bone quality. There are multiple hypotheses of how exactly RA destroys cartilage and leads to disruption of bone matrix and early osteoporosis but most hypothesize a link to inflammation [76]. Furthermore, the use of medications, especially corticosteroids, exacerbate the problem [77]. Even though there is theoretical concern of bone ingrowth, Armstrong and Whiteside (1991) evaluated cementless TKA in an older population with RA in the 1980s. Of 55 TKAs performed, 2 had supracondylar femur fractures and two had revisions due to infection and loosening. About 19% still had some level of pain at follow-up and average follow-up was only 44 months [78]. More recent studies demonstrate satisfactory clinical and radiographic results. At a short-term follow-up, Patel et al. (2018) showed no radiographic lucency, which short-term would be the time most concerning for initial difficulties with osseointegration [79]. At 15-year follow-up, survivorship has been shown to be greater than 95% [80,81].

### 3.4. Osteoporosis

Osteoporosis is common in patients awaiting total joint arthroplasty, with almost a quarter of patients with osteoporosis and almost double that with osteopenia [82]. Cementless TKA relies on osseointegration for fixation of the components and the quality of bone for that fixation has been a concern in patients with osteoporosis or osteopenia. A mechanical study compared 3 groups including a cemented tibial component, a cementless two-pegged tibial component, and a cementless keeled tibial component in “normal” and “osteoporotic” bone in the lab. The cemented component showed the least micromotion of the three designs and both cementless designs showed more micromotion in the “osteoporotic” bone compared to the “normal” bone [83]. Similarly, several studies identified that bone mineral density (BMD) had a significantly negative correlation with maximum total point motion (MTPM), indicating that poor bone quality was associated with more motion at the tibial component [84,85]. On the contrary, Linde et al. (2019) evaluated pre-operative systemic bone quality on tibial component migration in cemented NexGen stemmed tibial component (Zimmer Biomet) and cementless NexGen monoblock tibial component (Zimmer Biomet). At two years, there was no significant difference in maximum total point motion between osteoporotic and nonosteoporotic bone in either the cemented group or the cementless group, indicating bone quality may have a lesser effect on outcomes. However, the numbers were small in this study [86]. Similarly, patients with osteonecrosis, who also have poor bone quality, have shown excellent implant survivorship and clinical outcomes at minimum 3 year follow-up [87]. Therefore, patients with osteoporosis or poor bone quality may still be a candidate for cementless TKA, especially with modern designs.

## 4. Outcomes

It is known that cemented and cementless TKA both have good outcomes in regard to clinical, radiographic and survivorship. However, more interest in the literature has been placed to directly compare these fixation techniques, especially as more modern designs have shown better outcomes. As seen in Table 1, which illustrates literature published in the 21st century comparing cemented and cementless TKA in randomized controlled trials, there does not appear to be too much variation in outcomes between the cemented and cementless TKA.

### 4.1. Clinical & Radiographic Outcomes

Several studies looked at early clinical and radiographic outcomes between cemented and cementless TKA. At 2 year follow-up, Nam et al. (2019) showed no difference in post-operative Knee Society Score (KSS), Oxford Knee Score, Forgotten Joint Score, or overall satisfaction between the cemented and cementless TKAs of the same modern design. Similarly, there were no signs of component subsidence or progressive lucency on radiographs [13]. On the contrary, at 2 years, Fricka et al. (2015) did find radiolucencies in the cementless group as well as higher KSS (96.4 vs. 92.3) in the cemented versus cementless group, respectively [96]. Longer follow-up studies also showed higher KSS in the cemented group but essentially comparable results in other patient reported outcomes. Similarly, there were also equivalent results in regard to radiographic lucencies [97]. Most substantial, Liu et al. (2021) performed a systematic review and meta-analysis comparing outcomes of cemented and cementless TKA with 2–16 year follow-up. In total, there were 26 studies (11 randomized controlled trials), which included 2369 patients and 2654 patients in the cementless and cemented groups, respectively. Of these, 12 studies reported Knee Society knee score and found no significant difference between the cementless and cemented pooled data. However, the cementless group had better Knee Society Function scores than the cemented group in a pool of 9 studies (MD = 1.70, 95% CI [0.53, 2.86], *p* = 0.004). Furthermore, range of motion did not defer between the two groups even though there was significantly more manipulations under anesthesia in the cemented group [98]. Further long-term direct comparison studies are needed to compare survivorship between cemented and cementless TKA.

### 4.2. Complications & Survivability

Over the years, complications in both groups have decreased. Aseptic loosening is still a major cause of failure in both cementless and cemented TKA in addition to prosthetic joint infection. However, pooled data shows no significant difference between the two groups and only 2.9 and 2.4% of TKAs needing revision due to aseptic loosening in cementless and cemented TKAs, respectively [98]. Mercurio et al. (2022) found more aseptic loosening and manipulation under anesthesia in cemented TKA versus cementless TKA but did show less blood loss intraoperatively [97]. Similarly, survivorship has also improved over the years. Early on, cementless TKA had poor survivorship but changes in design changed these results [99]. At short-term follow-up, equivalent survivorship has been shown, with cementless failing due to instability and cemented failing due to infection [96]. Furthermore, even in earlier implants, a meta-analysis of long-term outcomes of cementless and cemented TKA showed no difference in 10-year survivorship (95.6% and 95.3%, respectively) [100]. Most substantial was the short and long-term analysis of the National Joint Registry, which included 96,519 TKAs. Ultimately, cemented TKA had better 10-year survivorship at 97% compared to 94.5% in the cementless group [101]. However, AJRR data reveals that cementless TKA outperformed cemented in the highest risk group, young males at 7 year follow-up [12].

### 4.3. Cost

As the number of cementless TKAs performed annually has increased the cost of the implants has decreased over time. The cost of cementless TKA implants ranges from $3047–$5116 compared to $2808–4750 for cemented TKA implants [14,102]. However, cementless TKA has lower operating room time cost and less supply costs. Most studies utilize conservative numbers in regard to estimating cost of the operating room and some fail to account opportunity cost or the ability to gain from that time savings [14,102]. Rassir et al. (2020) created a cost-effectiveness analysis to compare the two fixation types and patient reported outcomes within one year of surgery. Ultimately, inpatient and outpatient costs, costs per quality-adjusted-life year (QALY) and patient reported outcomes were similar between the two groups [103].

## 5. Conclusions

As designs have improved over time, the number of cementless TKAs performed has increased significantly particularly in the younger, more obese and more active patient. Comparative studies have shown good clinical and radiographic results between cementless and cemented TKA. This review has explored the evolution and current indications for cementless TKA. Today, orthopedic surgeons may consider increasing the use of cementless TKA, particularly in younger and larger populations. However, long-term studies comparing survivorship between cementless and cemented TKA are still needed. Cementless TKA may be the future and surpass cemented TKA as the gold standard pending future studies.

## Figures and Tables

**Table 1:** Randomized controlled trials comparing cemented and cementless TKA.

Study	Year	Mean Age Year (SD)	Gender (Male %)	Mean BMI kg/m^2^ (SD)	Final Number of TKA in Analysis	Implant	Follow-Up (Months)	Main Findings
Linde et al. [88]	2022	65.8 (5.4) vs. 67.2 (5.9)	50 vs. 50	28.3 (4.0) vs. 30.1 (5.7)	25 vs. 26	Vanguard vs. Regenerex (Zimmer Biomet)	24	-No significant difference in PROMs -Cementless had higher mean subsidence and greater variation at 1 year; had more radiolucent lines along the medial tibia at 6 months (*p* < 0.001) but decreased at 2 years (0.02)
van Ooij et al. [89]	2020	70.9 (6.6) vs. 64.6 (7.7)	34 vs. 52	29.6 (3.6) vs. 29.2 (3.4)	29 vs. 29	Advanced Coated System (Implantcast GmbH)	24	-No significant difference in PROMs -No significant difference in MTPM between 12–24 months -No significant difference in MTPM of the femoral component at 6 months but significantly more motion of the tibial component in the cementless group -2 cementless TKA required early revision due to septic loosening
Hasan et al. [39]	2020	66 (6.3) vs. 65 (6.7)	53 vs. 51	30 (3.1) vs. 28 (3.1)	34 vs. 35	Triathlon vs. 3D printed Tritanium (Stryker)	24	-No significant difference in PROMs -1 revision in cementless group due to tibial migration -Cementless TKA had higher MTPM, most substantial within the first 3 months
Nam et al. [13]	2019	63 (7.6) vs. 61.3 (7.0)	48 vs. 52	31.3 (4.7) vs. 31.1 (5.2)	65 vs. 76	Triathlon (Stryker)	24	-No significant difference in PROMs -1 revision in cemented group due to infection -No radiographic evidence of subsidence or loosening in either group -Cementless lower operative time (82.1 ± 16.6 compared with 93.7 ± 16.7 min, *p* = 0.001)
Fricka et al. [90]	2019	58.4 vs. 59.8	30 vs. 37	31.9 vs. 31.4	44 vs. 41	NexGen (Zimmer Biomet)	60	-No significant difference in PROMs and 95% both groups were satisfied -Equivalent survivorship (95.9% CI 90.3–100 and 95.3% CI 88.9–100, *p* = 0.98) w/2 revisions in the cemented group (infection, traumatic dislocation) and the cementless group (aseptic loosening, periprosthetic fracture) -At 5 years, no significant difference in radiolucency between groups
Van Hamersveld et al. [91]	2017	65.7 (6.3) vs. 66.8 (9.1)	57 vs. 37	28.6 (3.6) vs. 28.0 (3.3)	28 vs. 26	Triathlon (Stryker)—peri-apatite coated cemetless	60	-1 cemented revision due to ligament instability -MTPM significantly greater in cementless group at 5 years but cemented group had greater MTPM between 3 months and 5 years
Choy et al. [92]	2014	69 (6.8) vs. 65 (5)	8 vs. 8	29 (4) vs. 30 (6)	86 vs. 82	Low Contact Stress Rotating Platform (Depuy)	97	-No significant difference in PROMS, ROM, or radiographic measures -Radiolucent line noticed in 8% cemented and 13% cementless group -100% survival at minimum of 8 year follow-up
Kim et al. [93]	2014	54.3 vs. 54.3 (all less than 55 yo)	21 vs. 21	27.8 vs. 27.8	80 vs. 80 (simultaneous bilateral knees, one cemented and one cementless)	NexGen (Zimmer Biomet)	192	-No significant difference in PROMS, ROM, or satisfaction -No osteolysis in either group -1 infection in each group -Significantly more blood loss in cementless group -Survivorship 100% cemented vs. 98.7% cementless
Baker et al. [94]	2007	70 vs. 71	45 vs. 42	NA	277 vs. 224	Press-Fit Condylar (Johnson & Johnson)	180	-Survival ascertained at mean follow-up of 8.9 years and at 15 years, survival rate was 80.7% in cemented and 75.3% in cementless -Total of 54 (10.8%) required further surgery, 9.7% in the cemented group 12.1% in the cementless group -26 revisions were due to aseptic loosening (5 cemented, 8 cementless)
Beaupré et al. [95]	2007	63.9 (5.8) vs. 62.9 (6.4)	37 vs. 39	NA	37 vs. 33	Scorpio Tibia component (Stryker)	60	-No significant difference in PROMS, ROM, or radiographic measures -1 cemented and 2 cementless required manipulation under anesthesia for loss of flexion -No patient required revision surgery for tibia component in either group

All columns comparing cemented vs. cementless, respectively. PROMs = patient-reported outcome measures; MTPM = maximum total point motion (by radiostereometric analysis); ROM = range of motion; NA = not available.

## Data Availability

Not applicable.
